# Supplementary material for: Rapid turnover of life-cycle-related genes in the brown algae
Source: Genome Biol. 2019 Feb 14;20:35. doi: 10.1186/s13059-019-1630-6 (PMC6374913; doi:10.1186/s13059-019-1630-6)
Supplement: Supplementary file 3 — Protein alignments, corresponding Gblocks html file and CDS sequences for the lineage conserved gametophyte-biased genes. (ZIP 2109 kb) [file 13059_2019_1630_MOESM3_ESM.zip › Con_GA_alignments/Ec-20_001410.aa_ali.fasta-gb.htm]

Ec-20\_001410.aa.fasta.trans.aa\_ali.fasta


## Gblocks 0.91b Results

Processed file: **Ec-20\_001410.aa.fasta.trans.aa\_ali.fasta**  
Number of sequences: **2**  
Alignment assumed to be: **Protein**  
New number of positions: **585** (selected positions are underlined in blue)

```
                         10        20        30        40        50        60
                 =========+=========+=========+=========+=========+=========+
Ec-20_001410     MSETESLAELVVNSVTKSALLRSASDSSSLVKTGNGSMNGAGSCIVGDEVEPAVEAAAAV
MSTRG.49565.1    ------------------------------------------------------------
                                                                             


                         70        80        90       100       110       120
                 =========+=========+=========+=========+=========+=========+
Ec-20_001410     AVHSHERPATTQSGLMSETESLAELVVNSVTRSALLRSIPSNNSLIKTGANSIHGTGNFP
MSTRG.49565.1    ------------------------------------------------------------
                                                                             


                        130       140       150       160       170       180
                 =========+=========+=========+=========+=========+=========+
Ec-20_001410     IEHELEASAAALKADAHEPPASTKSVLMSEMESLAELVANSVTKSALLGSASDSSSLIKT
MSTRG.49565.1    ------------------------------------------------------------
                                                                             


                        190       200       210       220       230       240
                 =========+=========+=========+=========+=========+=========+
Ec-20_001410     GNGSMNGTGSGIIEYEVEPAAAAPAVAVHSHERPATTQSGLMSETESLAELVVNSVTNSA
MSTRG.49565.1    ------------------------------------------------------------
                                                                             


                        250       260       270       280       290       300
                 =========+=========+=========+=========+=========+=========+
Ec-20_001410     LLRSIPSNNSLIKTRHGSMHGTGNVAVEHELEASTTAPVVDAHELPASIKSSLMSETESL
MSTRG.49565.1    ------------------------------------------------------------
                                                                             


                        310       320       330       340       350       360
                 =========+=========+=========+=========+=========+=========+
Ec-20_001410     AELLVNSVTQSALLRSTSDNSSLIKAGHGSMNGTGSGIIEDEVEPAAAASVVVTDERPSS
MSTRG.49565.1    ------------------------------------------------------------
                                                                             


                        370       380       390       400       410       420
                 =========+=========+=========+=========+=========+=========+
Ec-20_001410     TQLGLMSETESLAELVVHSVTTSAVLNFTPSDNSLIKDGVGSMNSVGSGAALNGVELAVE
MSTRG.49565.1    ------------------------------------------------------------
                                                                             


                        430       440       450       460       470       480
                 =========+=========+=========+=========+=========+=========+
Ec-20_001410     ATLNAENQAAPVKNSVPDEAETLTGITVQDVAASVLQNSTGSAFKASKESGPLSRSSVGK
MSTRG.49565.1    ------------------------------------------------------------
                                                                             


                        490       500       510       520       530       540
                 =========+=========+=========+=========+=========+=========+
Ec-20_001410     WVTPRKDGDTESSKFISNGPNNRCRKRSRDDAGSNQTNRKNATASEAGVGRKNHKRTGAT
MSTRG.49565.1    ------------------------------------------------------------
                                                                             


                        550       560       570       580       590       600
                 =========+=========+=========+=========+=========+=========+
Ec-20_001410     YTDHIGSSNSRRLEGPGDAGGAGGDHLAVRKRDQEYSSGTFEEDDSVAVNPNGSILTDNR
MSTRG.49565.1    ------------------------------------------------------------
                                                                             


                        610       620       630       640       650       660
                 =========+=========+=========+=========+=========+=========+
Ec-20_001410     SQRPNAYQRSRKNLPSGGRSVVSNATSAFSSSPPTPTRSPQGSIGCRGPRHNRASTLQGT
MSTRG.49565.1    ------------------------------------------------------------
                                                                             


                        670       680       690       700       710       720
                 =========+=========+=========+=========+=========+=========+
Ec-20_001410     GKVRSSAVVPRVHDDHLKSPASQTRTGNGKDKTVDPGSPVSGRPVAQGVAPFPSSASMVS
MSTRG.49565.1    ------------------------------------------------VAVFVFWTSNIS
                                                                 ############


                        730       740       750       760       770       780
                 =========+=========+=========+=========+=========+=========+
Ec-20_001410     ARETSPSDPANSTPTDRTNGKPGSPAGAATVPPVVAPVERSACGRSGSVGKVVGLAGLKH
MSTRG.49565.1    Q-------GGASSTAEITTGKRVEVSGATLTQRQKQDLER--------------------
                            #################                                


                        790       800       810       820       830       840
                 =========+=========+=========+=========+=========+=========+
Ec-20_001410     DVTAWFDDEVLAPSATRVVATVAKNRGGRPASRLSSTSRTTGILHDAPAAYKDSGKTIFS
MSTRG.49565.1    ------------------------------------------------------------
                                                                             


                        850       860       870       880       890       900
                 =========+=========+=========+=========+=========+=========+
Ec-20_001410     NCSSTRNSRCGSLDVDVGIGQHEPGLETVGDGTPKPEEHGNRQHQTVHPARLFHLLWQRD
MSTRG.49565.1    -----------------------------QNGSHHCDRRDHRQHQ---------------
                                                                             


                        910       920       930       940       950       960
                 =========+=========+=========+=========+=========+=========+
Ec-20_001410     RSRCPFSRCPIPDTVVFNSMGEAGDWFFTSKREGGVKRKLTSNTLFSNIFREFCKGSTDG
MSTRG.49565.1    --------------------------------RGTVIRHFTARELHSFLF----GGNRD-
                                                  #################          


                        970       980       990      1000      1010      1020
                 =========+=========+=========+=========+=========+=========+
Ec-20_001410     DPVAVIISWIPRNDDGIVSVTSAGVGRDIQPGMEDARSQQHLQKPDSPQPQHAKFHHPQL
MSTRG.49565.1    ---------YHKLSTGATSHAASAAGTDGS------------------------------
                                #############                                


                       1030      1040      1050      1060      1070      1080
                 =========+=========+=========+=========+=========+=========+
Ec-20_001410     ENQQDAAIRHLTARELHDFLFGSNHHQHPVPPVVETATGGKQQRNEAFNGDEVDAFHTIG
MSTRG.49565.1    -KQWQTNARFKTKTTVHHFRKTRSDNEYRAHPLARNHGGADFK-----------------
                   #################                                         


                       1090      1100      1110      1120      1130      1140
                 =========+=========+=========+=========+=========+=========+
Ec-20_001410     RGSLLHLQARSDGGTGTYTETFARRHIERSEAAATAAGAGAGDRDSGGWCLLQQYIGPTG
MSTRG.49565.1    ---------------WPHTETFGPRNSERSEAAARAAGEGAGDDSGGGWCLLQRFIAPAG
                                   ##########################################


                       1150      1160      1170      1180      1190      1200
                 =========+=========+=========+=========+=========+=========+
Ec-20_001410     TRNSTLRCHWSALSSHTPVR---------VRSAHPDVVERHRFRLGSVEEIEMSFRERSP
MSTRG.49565.1    SKVSSLRCDWTASPSRRPPRPSTSVDAHAASGITPAAGARGLPRWGSVEEVDTSFRERSP
                 ####################              ##########################


                       1210      1220      1230      1240      1250      1260
                 =========+=========+=========+=========+=========+=========+
Ec-20_001410     DRSPFRQRLSDQS-------------VPKQSVKETAELELGAAGRLSSSGGVYNGCLLER
MSTRG.49565.1    ARSPFRERPVERSGTMLPENPQHRLPTSSHSAKGVAAREHGAAGRISTSERVSEGCVLQR
                 #############                 ##############################


                       1270      1280      1290      1300      1310      1320
                 =========+=========+=========+=========+=========+=========+
Ec-20_001410     STNVFRADRKDLPCEHRCVTFEEPPPSSRGGLWPRSVREPMKSPALRQTVVEACTAALDH
MSTRG.49565.1    CTNIFRADRRDLTCEDRCVTFEEPAPSSRGGLWPRSEKGPVRSSPLRQTLVEGCSAISDH
                 ############################################################


                       1330      1340      1350      1360      1370      1380
                 =========+=========+=========+=========+=========+=========+
Ec-20_001410     VSLVTRGEFRPKLATSYFRQGEDGRTFLMFLSRWGQSPSLGIDANEPLLCRTGTGSATLP
MSTRG.49565.1    IHQVTKGVFRVQAATAFFRQGADGRTYLMFLSTWGESPTIGIDRFGPLLAWATN--HSLA
                 #################################################         ##


                       1390      1400      1410      1420      1430      1440
                 =========+=========+=========+=========+=========+=========+
Ec-20_001410     IHDSVGLDNQARLQHVLGIGVEP------TVATTISPDREGSVGGLGALQEYGRPVHHGL
MSTRG.49565.1    EADVEGHEVEAQRQHKMSSLNEQGHTRVMEERTRRPPEPGGHGQGDGELPFRSAASISER
                 ######################          #################           


                       1450      1460      1470      1480      1490      1500
                 =========+=========+=========+=========+=========+=========+
Ec-20_001410     VCPGCGRSGIRGGVRSDNNGSSTMHGDTQVRGIAPSHLVMTQNTGPAMCCPSANCNNAVA
MSTRG.49565.1    VCPGCGCPGVRGGVRR-IDGEPTICDKASTRGRTAQRDGLQV------AEG-TARNVAIE
                 ###############    #############                       #####


                       1510      1520      1530      1540      1550      1560
                 =========+=========+=========+=========+=========+=========+
Ec-20_001410     NLGDCTGNDKVAAGEESSSRAPSLHGLASTTFSPLDRTVYTSLGIVVDVCTWSETLRGGH
MSTRG.49565.1    RPGASAGSCENVSKTPPWLRLPSAGSSVSTTSSAVRGAFHTSLGVIADVCTSRSQPDAPC
                 #######            ################################         


                       1570      1580      1590      1600      1610      1620
                 =========+=========+=========+=========+=========+=========+
Ec-20_001410     AARNGDCGSIANNGRRTIARIGVKRCFRVLNRLHPGVRAEAISVRCNEDLRLRLRRVSVC
MSTRG.49565.1    QPKVTHFQGAAWPRDSARTPPGFERCLRVLGRIYPGVPAKTIAERCNEDLRLRLREVSVC
                                      #######################################


                       1630      1640      1650      1660      1670      1680
                 =========+=========+=========+=========+=========+=========+
Ec-20_001410     RECALEIAGASDVRLREMNDAAKRKMTELAARLGVTTSAPPLSERQRKARVSEDIKAFQR
MSTRG.49565.1    RECAVEINGASGARLKALDDAAKSRMGELAGRLGIMSGQTSLSVRQRKACVMEDIKAFRH
                 ############################################################


                       1690      1700      1710      1720      1730      1740
                 =========+=========+=========+=========+=========+=========+
Ec-20_001410     RVASASSEPSQTRTRLQNCFGGGPSRMGEVPIKQFGYVPKRPLGKRLGNRPSTTDGLSPR
MSTRG.49565.1    RITSGSSASCPAETHRNRCFGGGPSRIHESSVKHSGHLPRRPLGPKC-IRPRTTDGISPR
                 ############################################     ###########


                       1750      1760      1770      1780      1790      1800
                 =========+=========+=========+=========+=========+=========+
Ec-20_001410     QQVFQHSPRKIVSATQPPGGFSVATKYHEEVRMATPSNGKSATETSFRNNNEGRANETTT
MSTRG.49565.1    QQ-HDHCSRKIVSA----------------------------------------------
                 ##                                                          


                       1810      1820      1830      1840      1850      1860
                 =========+=========+=========+=========+=========+=========+
Ec-20_001410     SSALRHTSCGSDADGGSGGDGGGGGGGDGDSNEDRVGPDDGFNSSNECVGADESSTMNAW
MSTRG.49565.1    ------------------------------------------------------------
                                                                             


                       1870      1880      1890      1900      1910      1920
                 =========+=========+=========+=========+=========+=========+
Ec-20_001410     QDERERHIQTRHEHKQQRQRQRQQQQQPAHTGTSGWNFYDRSNVAFRSRRLFAAYSGTGI
MSTRG.49565.1    ------------------------------------------------------------
                                                                             


                       1930      1940      1950      1960      1970      1980
                 =========+=========+=========+=========+=========+=========+
Ec-20_001410     GDPGGGGVSPRRAGNARCAKASPAEDGAGARDVSAPPPRALRVALRPSNGRRQSARKMLR
MSTRG.49565.1    ------------------------------------------------------------
                                                                             


                       1990      2000      2010      2020      2030      2040
                 =========+=========+=========+=========+=========+=========+
Ec-20_001410     RWEAALNIRHWSPVLRDGILMTGQVAETVSRKNLGSGARTATENPAIRAAMYVAAELDLV
MSTRG.49565.1    ------------------------------------------------------------
                                                                             


                       2050      2060      2070
                 =========+=========+=========+=========
Ec-20_001410     VNAATARMPREVGGGVNQRVSRRGEPNAGRERLLPGTAT
MSTRG.49565.1    ---------------------------------------
```

```
Parameters used
Minimum Number Of Sequences For A Conserved Position: 2
Minimum Number Of Sequences For A Flanking Position: 2
Maximum Number Of Contiguous Nonconserved Positions: 8
Minimum Length Of A Block: 10
Allowed Gap Positions: None
Use Similarity Matrices: Yes
```

```
Flank positions of the 16 selected block(s)
Flanks: [709  720]  [732  748]  [934  950]  [976  988]  [1023  1039]  [1099  1160]  [1175  1213]  [1231  1369]  [1379  1402]  [1413  1429]  [1441  1455]  [1460  1472]  [1496  1507]  [1520  1551]  [1582  1724]  [1730  1742]  

New number of positions in Ec-20_001410.aa.fasta.trans.aa_ali.fasta-gb:  585  (28% of the original 2079 positions)
```
